# Fine mapping of two grain chalkiness QTLs sensitive to high temperature in rice

**DOI:** 10.1186/s12284-021-00476-x

**Published:** 2021-04-01

**Authors:** Weifeng Yang, Jiayan Liang, Qingwen Hao, Xin Luan, Quanya Tan, Shiwan Lin, Haitao Zhu, Guifu Liu, Zupei Liu, Suhong Bu, Shaokui Wang, Guiquan Zhang

**Affiliations:** Guangdong Provincial Key Laboratory of Plant Molecular Breeding, State Key Laboratory for Conservation and Utilization of Subtropical Agro-Bioresources, South China Agricultural University, Guangzhou, 510642 China

**Keywords:** Grain chalkiness, Grain quality, Single-segment substitution line, Substitution mapping, Heat stress, Rice

## Abstract

**Background:**

Grain chalkiness is one of important factors affected rice grain quality. It is known that chalkiness is affected by the high temperature during the seed filling period. Although a larger of QTLs for chalkiness were reported across all 12 chromosomes, only a few of the QTLs were fine mapped or cloned up to now. Here, we fine map two QTLs for chalkiness in two single-segment substitution lines (SSSLs), 11–09 with substitution segment from *O. sativa* and HP67–11 with substitution segment from *O. glaberrima.*

**Results:**

The grain chalkiness of SSSLs 11–09 and HP67–11 was significantly lower than that in the recipient Huajingxian 74 (HJX74) in consecutive 8 cropping seasons. The regression correlation analysis showed that percentage of chalky grain (PCG) and percentage of chalky area (PCA) were significantly and positively correlated with percentage of grain chalkiness (PGC). Two QTLs for grain chalkiness were located on two chromosomes by substitution mapping. *qPGC9* was mapped on chromosome 9 with an estimated interval of 345.6 kb. *qPGC11* was located on chromosome 11 and delimited to a 432.1 kb interval in the *O. sativa* genome and a 332.9 kb interval in the *O. glaberrima* genome. *qPGC11* is a QTL for grain chalkiness from *O. glaberrima* and was mapped in a new region of chromosome 11. The effect of two QTLs was incomplete dominance. The additive effects of two QTLs on chalkiness in second cropping season (SCS) were significantly greater than that in first cropping season (FCS).

**Conclusions:**

*qPGC11* is a new QTL for grain chalkiness. The two QTLs were fine mapped. The donor alleles of *qPGC9* and *qPGC11* were sensitive to the high temperature of FCS.

**Supplementary Information:**

The online version contains supplementary material available at 10.1186/s12284-021-00476-x.

## Background

Rice is a staple food that provides at least 20% of the daily caloric needs of more than half of the world’s people. To meet the demands of a growing global population, rice breeders have been aiming at the high yield of varieties (Peng et al. 2004). In recent years, with the improvement of people’s living standards, people pay more and more attention to rice quality. Rice varieties were required to have both a higher grain yield and a better grain quality. Rice quality includes the appearance quality, processing quality, nutritional quality, cooking and eating quality, and so on (Gao et al. 2016). Grain chalkiness not only affects the grain appearance, but also has adverse effects on milling and cooking properties. Varieties with higher head rice yield, higher transparency and less chalkiness are more popular in the market (Sreenivasulu et al. 2015; Misra et al. 2019).

Chalkiness is one of variable parameters that influence grain quality. It is defined as the opaque part of translucent endosperm in grains. In the early and middle stages of seed development, the occurrence of temperature stress causes uneven seed filling and storage biosynthesis obstacles, leading to the formation of chalkiness. The chalkiness is affected by the high temperature during the seed filling period (Masutomi et al. 2015; Sreenivasulu et al. 2015; Morita et al. 2016). Exposure to high temperature during the entire storage phase resulted in triggering of > 90% of chalky grains. When exposed during the early storage phase, nearly half of the grains had the chalky phenotype, while a much lower frequency was shown in the exposure during middle and late phases of storage (Ishimaru et al. 2019). The chalkiness of rice varieties varied greatly. Variety surveys showed that the proportion of chalky grains of newly developed varieties was higher than that of the old modern varieties, and the proportion of chalky grains of the hybrid varieties was higher than that of other modern varieties (Laborte et al. 2015). Therefore, high-yielding varieties usually have higher chalkiness levels (Misra et al. 2019).

Grain chalkiness is a complex polygenic quantitative trait (Sreenivasulu et al. 2015). Percentage of grain chalkiness (PGC) is a quantitative index of chalkiness phenotype (Misra et al. 2019). More than one hundred of QTLs for the chalkiness trait were reported across all 12 chromosomes (Sreenivasulu et al. 2015). A larger number of the QTLs for chalkiness were detected on chromosomes 5, 6 and 8 in different genetic backgrounds and environments. The QTL clusters were found on the hotspot regions of the three chromosomes (He et al. 1999; Tan et al. 2000; Wan et al. 2005; Hao et al. 2009; Chen et al. 2011; Guo et al. 2011; Liu et al. 2011; Liu et al. 2012; Li et al. 2014; Peng et al. 2014; Zhao et al. 2015; Chen et al. 2016; Gao et al. 2016; Yun et al. 2016; Zhao et al. 2016; Wang et al. 2017; Zhu et al. 2018a; Misra et al. 2019; Misra et al. 2020). Some QTLs were found to be related with chalkiness under high temperature stress (Nevame et al. 2018). Kobayashi et al. (2007) detected three QTLs for chalkiness under high temperature stress in *japonica* varieties. Tabata et al. (2007) identified four QTLs for chalkiness in a RIL population derived from a cross between a heat stress-tolerant variety and a heat stress-sensitive variety. Wada et al. (2015) and Miyahara et al. (2017) identified a set of QTLs for chalkiness under heat stress condition using a RIL population derived from a cross between a heat-tolerant variety and a heat stress-sensitive variety, and validated the effect of two QTLs *qMW4.1* and *qWB8* under heat stress during the ripening period. However, only a few QTLs for chalkiness were fine mapped or cloned up to now. *Chalk5* on chromosome 5 is the firstly cloned QTL for chalkiness, which encodes a vacuolar H^+^-translocating pyrophosphatase (V-PPase) with inorganic pyrophosphate (PP_i_) hydrolysis and H^+^-translocation activity (Li et al. 2014).

Like near-isogenic lines (NILs), chromosome single-segment substitution lines (SSSLs) carry only one substitution segment from donors in the recipient genetic background (Zhang et al. 2004; Keurentjes et al. 2007; Zhang 2019). We have developed a library of 2360 SSSLs, which were derived from 43 donors of 7 species of rice AA genome in the genetic background of Huajingxian 74 (HJX74), an *indica* elite variety in southern China (Zhang et al. 2004; Xi et al. 2006; Zhang 2019). These SSSLs were widely used to detect QTLs for complex traits (Zhang et al. 2012; Zhu et al. 2014; Yang et al. 2016; Zhou et al. 2017; Zhu et al. 2018b), to clone QTLs of agronomic importance and to mine alleles of different functions (Zeng et al. 2006; Teng et al. 2012; Wang et al. 2012; Fang et al. 2019; Sui et al. 2019). Recently, the SSSLs were used to detect QTLs controlling stigma exsertion rate (SER) of rice. A total of 11 QTLs for SER were mapped on 6 chromosomes of rice genome (Tan et al. 2020; Tan et al. 2021). In present study, two QTLs for grain chalkiness on chromosomes 9 and 11, *qPGC9* and *qPGC11*, were fine mapped. Both QTLs were sensitive to high temperature. Fine mapping of the two QTLs laid a foundation for cloning the genes and revealing the mechanism of chalkiness heat stress.

## Results

### Grain chalkiness in SSSLs

**Fig. 1:**
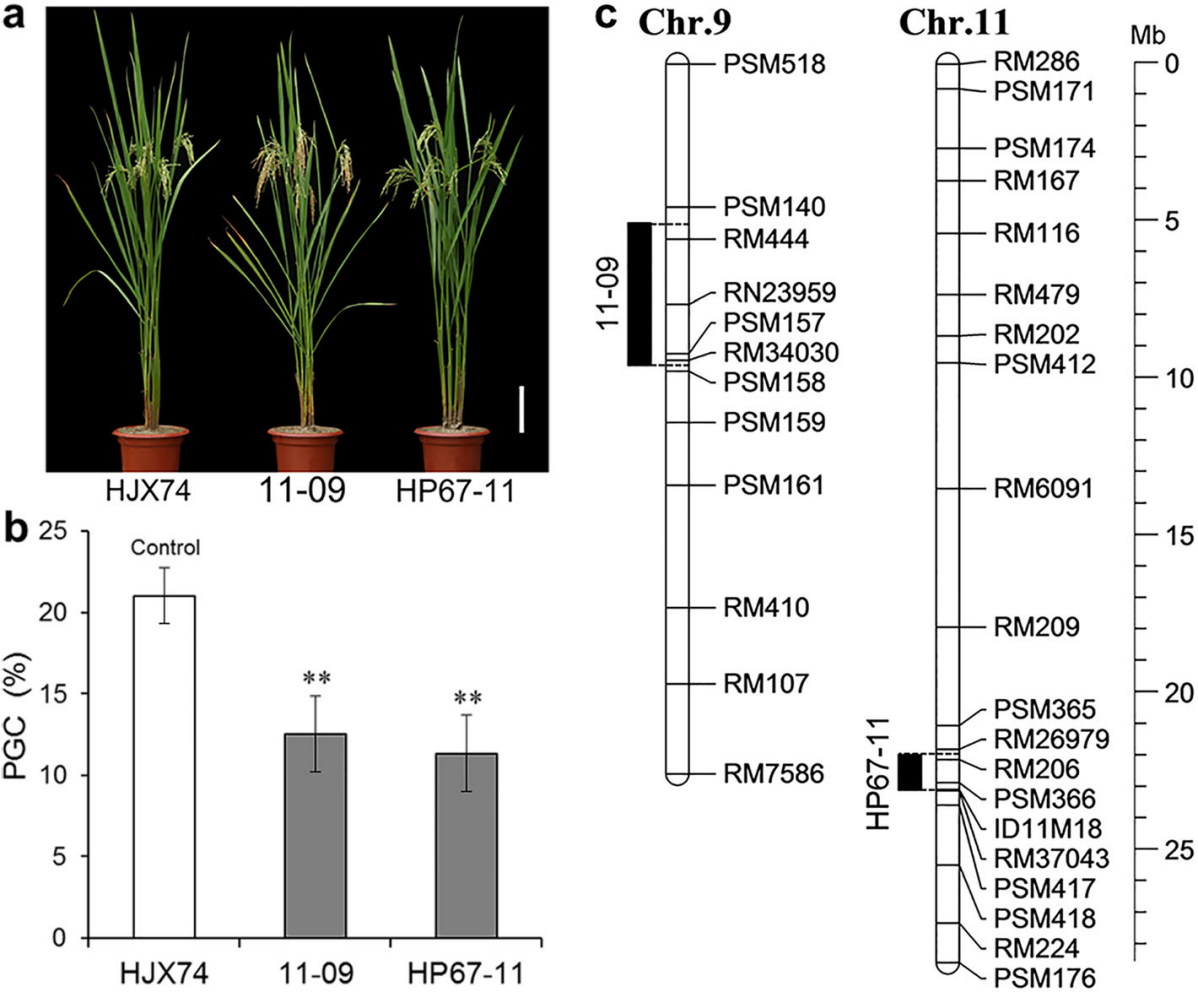
Grain chalkiness in HJX74 and SSSLs. **a**, Plant type of HJX74 and SSSLs 11–09 and HP67–11. Scale bar: 15 cm. **b**, Percentage of grain chalkiness (PGC) (%) in HJX74 and SSSLs. Data were presented as mean ± S.E. of eight cropping seasons. One-way ANOVA, two-tailed, Dunnett *t*-test was used to generate the differences. **c,** Chromosome locations of the two SSSLs. Physical distance (Mb) is shown as rulers on the right of chromosome. Black bars on the left of each chromosome represent the estimated length of substitution segments in the SSSLs. *Chr.* chromosome, *Mb* megabase

Two SSSLs 11–09 and HP67–11 with lower grain chalkiness were selected from the HJX74-SSSL library (Fig. 1). The SSSLs were used to investigate grain chalkiness in consecutive 8 cropping seasons from the first cropping season (FCS) of 2016 to the second cropping season (SCS) of 2019. On average, the percentage of grain chalkiness (PGC) of 11–09 and HP67–11 was 12.5% and 11.3% respectively, which was significantly lower than 21.0% of recipient HJX74, respectively (Fig. 1b and Additional file 1: Table S1). This meant that the two SSSLs each carried a QTL for PGC in their substitution segments.

The substitution segments of 11–09 and HP67–11 were surveyed by densifying molecular markers (Additional file 1: Table S2). The estimated length of substitution segments was 4531.2 kb in 11–09 and 1135.4 kb in HP67–11 (Fig. 1c and Additional file 1: Table S3).

Eight agronomic traits of 11–09 and HP67–11 were investigated. All traits of HP67–11 had no significant difference with HJX74. For 11–09, plant height, number of panicles per plant, total number of grains per plant and 1000-grain weight had no significant difference with HJX74, while heading date, grain length, grain width and grain weight per plant had significant difference with HJX74 in two cropping seasons or in only one cropping season (Fig. 1a and Additional file 1: Table S4). The results showed that the genetic background of SSSLs was similar to that of HJX74 except for grain chalkiness.

### Substitution mapping of *qPGC9* for grain chalkiness

**Fig. 2:**
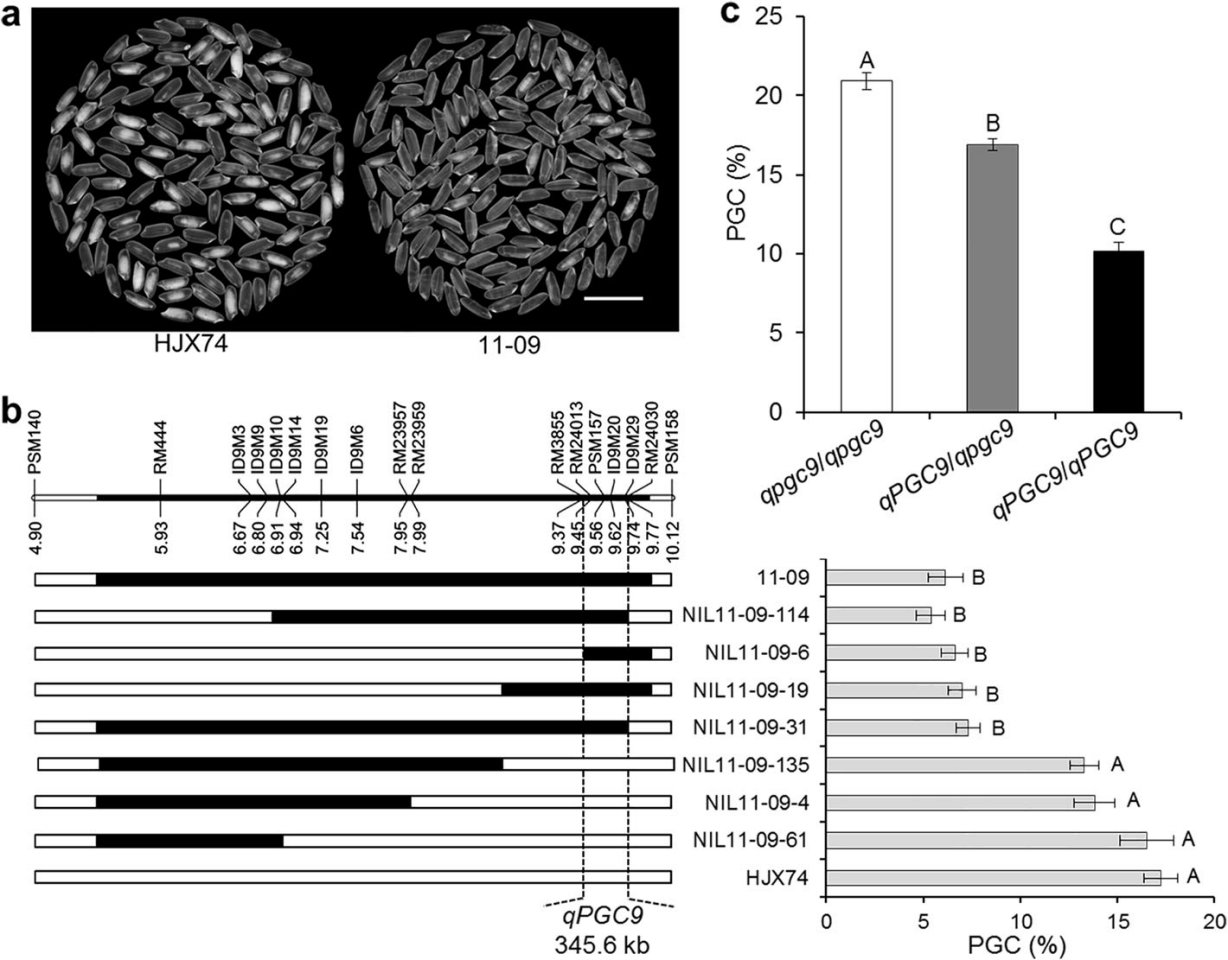
Substitution mapping of *qPGC9* for grain chalkiness***.*** **a**, The head rice appearance of the HJX74 and 11–09. Scale bar: 1 cm. **b**, Substitution mapping of *qPGC9*. The positions of substitution segments and the PGC of 11–09 and its NILs are shown, with HJX74 as the control. The numbers under the chromosome are physical distance (Mb). White and black blocks represent the homozygous genotypes of HJX74 and 11–09, respectively. **c**, PGC effects of three genotypes of *qPGC9* in an F_2_ population. *qpgc9*/*qpgc9* represents homozygous genotype of HJX74 (*n* = 38); *qPGC9*/*qpgc9* represents heterozygous genotype of HJX74/11–09 (*n* = 68); *qPGC9*/*qPGC9* represents homozygous genotype of 11–09 (*n* = 34). Significant difference analysis in **b** and **c** was by one-way ANOVA, Duncan, two-tailed. Values in the lines among different letters are different at 1% level of significance

To map the QTL for grain chalkiness on the substitution segment of 11–09, the SSSL was used to develop secondary SSSLs or NILs. Seven NILs were developed from an F_2:3_ population derived from the cross of HJX74/11–09. The seven NILs were then investigated for grain chalkiness. PGC levels of four NILs, NIL11–09-6, NIL11–09-19, NIL11–09-31 and NIL11–09-114, were as low as 11–09, while PGC levels of three NILs, NIL11–09-4, NIL11–09-61 and NIL11–09-135, were as high as HJX74. Substitution segments of the four NILs with low PGC overlapped in the region between markers RM3855 and RM24030, while substitution segments of other three NILs with high PGC located outside the region. These results indicated that the QTL for grain chalkiness, *qPGC9*, was located in the region between markers RM3855 and RM24030 with the estimated interval length of 345.6 kb (Fig. 2a-b).

Using PSM157 marker in *qPGC9* interval, Chi-square test was performed in 140 individuals of F_2_ population. The results showed that the segregation ratio of the three marker genotypes was 1:2:1 (χ^2^ = 0.34 < χ^2^_0.01,2_ = 9.21) (Additional file 2: Fig. S1). The effect of heterozygous genotype (*qPGC9*/*qpgc9*) was significantly lower than that of dominant homozygous genotype (*qPGC9*/*qPGC9*) and significantly higher than that of recessive homozygous genotype (*qpgc9*/*qpgc9*). The result showed that *qPGC9* was incomplete dominance (Fig. 2c).

### Substitution mapping of *qPGC11* for grain chalkiness

**Fig. 3:**
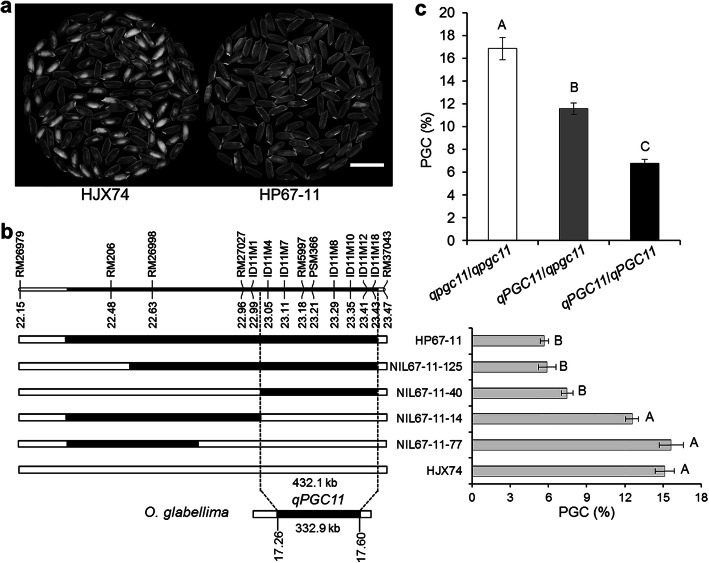
Substituted mapping of *qPGC11* for grain chalkiness. **a**, The head rice appearance of the HJX74 and HP67–11. Scale bar: 1 cm. **b**, Substitution mapping of *qPGC11*. The positions of substitution segments and the PGC of HP67–11 and its NILs are shown, with HJX74 as the control. The numbers under the chromosome are physical distance (Mb). White and black blocks represent the homozygous genotypes of HJX74 and HP67–11, respectively. **c**, PGC effects of three genotypes of *qPGC11* in an F_2_ population. *qpgc11*/*qpgc11* represents homozygous genotype of HJX74 (*n* = 26); *qPGC11*/*qpgc11* represents heterozygous genotype of HJX74/HP67–11 (*n* = 49); *qPGC11*/*qPGC11* represents homozygous genotype of HP67–11 (*n* = 30). Significance analysis in **b** and **c** was by one-way ANOVA, Duncan, two-tailed. Values in the lines among different letters are different at 1% level of significance

To map the QTL for grain chalkiness on the substitution segment of HP67–11, the SSSL was used to develop NILs. Four NILs were developed from an F_2:3_ population derived from the cross of HJX74/HP67–11. Grain chalkiness of the four NILs were then investigated. PGC levels of two NILs, NIL67–11-125 and NIL67–11-40, were as low as HP67–11, while PGC levels of two NILs, NIL67–11-14 and NIL67–11-77, were as high as HJX74. Substitution segments of the two NILs with low PGC overlapped in the region between markers ID11M1 and RM37043, while substitution segments of other two NILs with high PGC located outside the region. These results indicated that the QTL for grain chalkiness, *qPGC11*, was located in the region between markers ID11M1 and RM37043 with the estimated interval length of 432.1 kb (Fig. 3a-b). Because the substitution segment of HP67–11 was derived from *O. glaberrima*, the estimated length between markers ID11M1 and RM37043 on the substitution segment was 332.9 kb in the *O. glaberrima* genome (Fig. 3b).

In the F_2_ population of 105 individuals, PSM366 marker in *qPGC11* region showed that the segregation ratio of the three marker genotypes was 1:2:1 (χ^2^ = 0.77 < χ^2^_0.01,2_ = 9.21) (Additional file 2: Fig. S2). The effect of heterozygous genotype (*qPGC11*/*qpgc11*) was significantly different from that of the homozygous genotypes (*qPGC11*/*qPGC11* and *qpgc11*/*qpgc11*). The result showed that *qPGC11* was incomplete dominance (Fig. 3c).

### Effects of percentage of chalky grain (PCG) and percentage of chalky area (PCA) on PGC

**Fig. 4:**
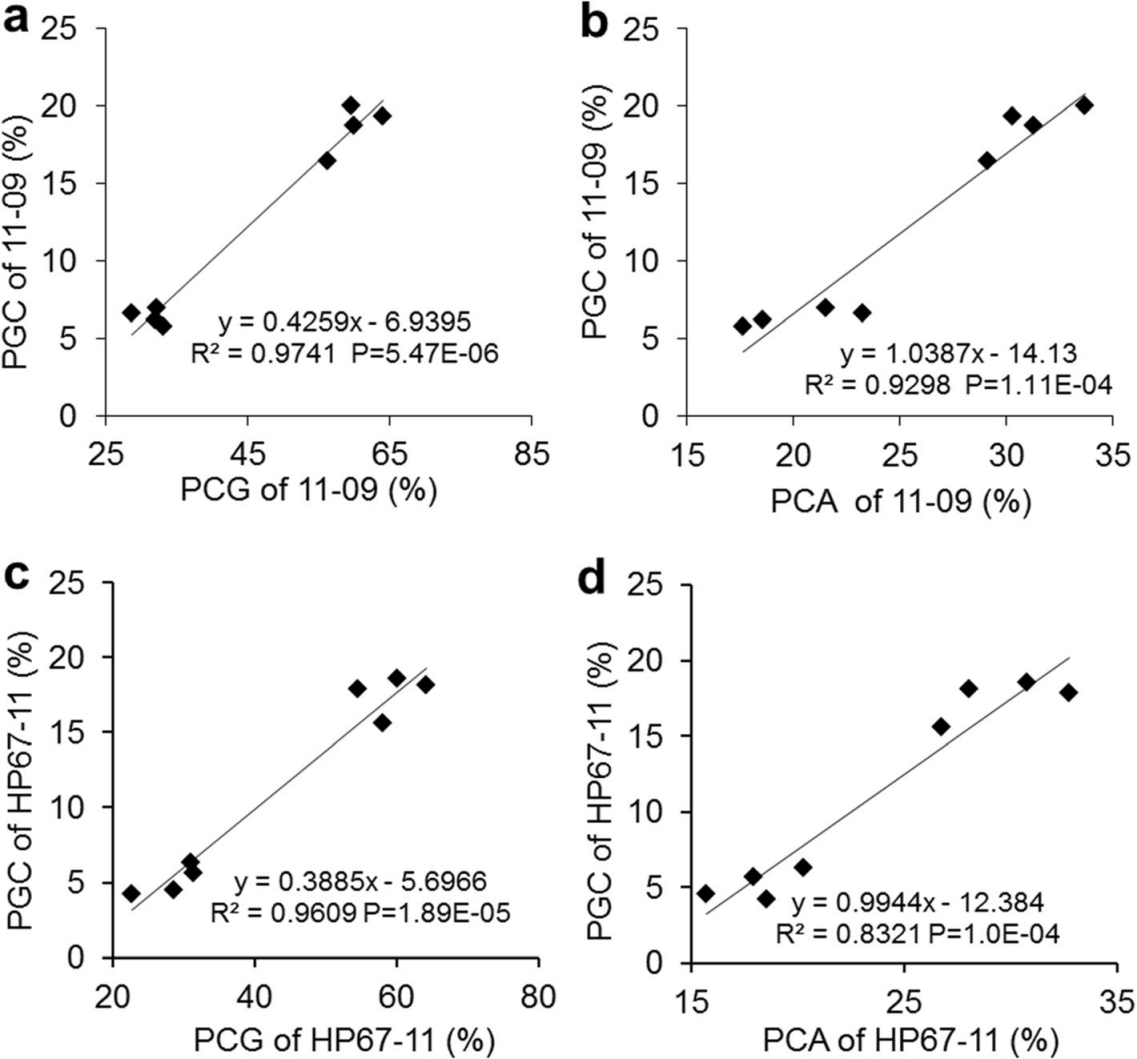
Regression correlation analysis between PCG and PGC and between PCA and PGC of SSSLs 11–09 and HP67–11*.* R^2^ represents the percentage of x contribution to y phenotype variation. *PCG* percentage of chalky grain*, PCA* percentage of chalky area, *PGC* percentage of grain chalkiness

PGC is a quantitative index of grain chalkiness. PGC can be decomposed into PCG and PCA. The regression correlation analysis showed that PCG and PCA were significantly and positively correlated with PGC. Regression coefficients of PCG and PCA with PGC were 0.9741 and 0.9298 in 11–09 carrying *qPGC9*, and 0.9609 and 0.8321 in HP67–11 carrying *qPGC11*, respectively (Fig. 4). The results showed that both PCG and PCA had great contribution to PGC, and PCG contributed more to PGC than PCA.

### Effects of different cropping seasons on grain chalkiness

The grain chalkiness was tested in two cropping seasons per year. During flowering to harvest of rice, the day and night temperatures of FCS and SCS were very different. In 2016–2019, the average values of maximum temperature, minimum temperature and mean temperature were respectively 32.6 °C, 25.9 °C and 29.2 °C in FCS, and 29.0 °C, 21.5 °C and 25.3 °C in SCS. The mean temperature in SCS was 3.9 °C lower than that in FCS (Additional file 1: Table S5).

**Fig. 5:**
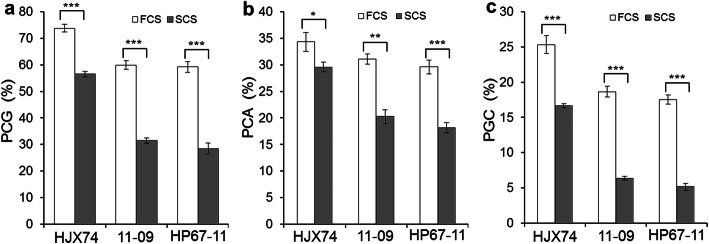
The difference of chalky traits between first cropping seasons (FCS) and second cropping seasons (SCS) in HJX74, 11–09 and HP67–11. *PCG* percentage of chalky grain (**a**). *PCA* Percentage of chalky area (**b**). *PGC* Percentage of grain chalkiness (**c**)

The values of PCG, PCA and PGC in FCS were significantly higher than those in SCS in all lines. The PGC of HJX74, 11–09 and HP67–11 was 25.3%, 18.7% and 17.5% in FCS, and 16.7%, 6.4% and 5.2% in SCS, respectively. The PGC of all lines in FCS was higher than that in SCS at 0.001 level of significance (Fig. 5). It is obvious that the PGC of HJX74 and SSSLs was greatly affected by higher temperature during the seed filling period.

### Additive effects of *qPGC9* and *qPGC11*

**Fig. 6:**
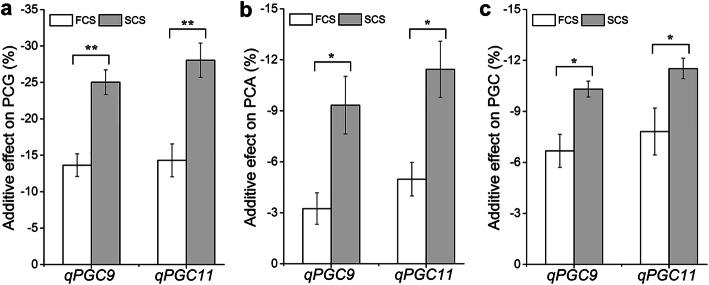
The additive effects of *qPGC9* and *qPGC11* on grain chalkiness in first cropping seasons (FCS) and second cropping seasons (SCS). *PCG* percentage of chalky grain (**a**). *PCA* Percentage of chalky area (**b**). *PGC* Percentage of grain chalkiness (**c**)

The effect of environment on grain chalkiness was reflected in the additive effect of the QTLs. According to the estimation of chalkiness phenotypes in 2016–2019, the additive effects of *qPGC9* and *qPGC11* on PCG, PCA and PGC in SCS were significantly greater than those in FCS. For *qPGC9*, the additive effects on PCG, PCA and PGC were − 13.6%, − 3.2% and − 6.7% in FCS, and − 25.0%, − 9.3% and − 10.3% in SCS, respectively. For *qPGC11*, the additive effects on PCG, PCA and PGC in FCS and SCS were − 14.3%, − 5.0% and − 7.8%, and − 28.0%, − 11.4% and − 11.5%, respectively (Fig. 6). The additive effects of *qPGC9* and *qPGC11* on chalkiness in SCS were almost twice of those in FCS. Obviously, the additive effects of the two QTLs on chalkiness were decreased by the high temperature of FCS.

## Discussion

### Fine mapping of the QTLs for grain chalkiness

More than one hundred of QTLs for chalkiness trait were reported across all 12 chromosomes. However, only a few QTLs for chalkiness were fine mapped up to now (Sreenivasulu et al. 2015; Misra et al. 2020). On chromosome 9, several QTLs for chalkiness were identified (Wan et al. 2005; Chen et al. 2011; Liu et al. 2011; Gao et al. 2016; Zhao et al. 2016; Misra et al. 2020). Wan et al. (2005) identified *qPGWC-9*/*qACE-9*/*qDEC-9* in the 1.8–10.8 Mb region of chromosome 9 with an interval about 9.0 Mb. Zhao et al. (2016) identified *qPGWC9b*/*qDEC9* in the 7.9–12.3 Mb region of chromosome 9 with an interval about 4.4 Mb. In this study, we fine mapped *qPGC9* on chromosome 9 in the 9.4–9.8 Mb region between markers RM3855 and RM24030 with the estimated interval length of 345.6 kb (Fig. 2). The mapping interval of *qPGC9* was much narrower than that of QTLs reported previously. On chromosome 11, several QTLs for chalkiness were detected (Chen et al. 2011; Peng et al. 2014; Zhao et al. 2016). In this study, HP67–11 carrying the substitution segment on chromosome 11 from *O. glaberrima* was used to detect QTL for chalkiness. *qPGC11* on the substitution segment was fine mapped in the 23.0–23.5 Mb region between markers ID11M1 and RM37043 with the estimated interval length of 432.1 kb in *O. sativa* genome and 332.9 kb in *O. glaberrima* genome (Fig. 3). This is the first QTL for chalkiness from *O. glaberrima*. The *qPGC11* is also new in its mapping region, in which no QTLs for chalkiness was reported previously. Fine mapping of *qPGC9* and *qPGC11* laid a foundation for gene cloning.

Grain chalkiness and grain shape are two important traits for rice grain quality. Grain width was positively correlated with chalkiness (Zhao et al. 2015). It was found that some QTLs controlling grain width were overlapped with chalk QTLs (Wang et al. 2017). Misra et al. (2020) showed that the phenotypic variation for chalkiness had a weak positive correlation with grain width. In this study, 11–09 showed significant difference with HJX74 in grain length and grain width in FCS of 2019, but no significant difference in SCS of 2018. HP67–11 had no significant difference with HJX74 in grain length and grain width in the two cropping seasons (Additional file 1: Table S4). To analyze the relationship of grain shape and grain chalkiness, the NILs used to map the QTLs for grain chalkiness (Figs. 2 and 3b) were used to investigate grain shape. The results showed that the grain length and grain width were significant different in the NILs but no linkage between grain shape and grain chalkiness of *qPGC9* (Additional file 1: Table S6), and were no significant different in the NILs of *qPGC11* (Additional file 1: Table S7). The results indicated that the grain chalkiness of *qPGC9* and *qPGC11* was no relationship with grain shape.

### Influence of high temperature on grain chalkiness

It is found that the high temperature during the seed filling period triggers non-uniform filling, leading to chalk formation (Masutomi et al. 2015; Sreenivasulu et al. 2015; Morita et al. 2016; Ishimaru et al. 2019). Some QTLs controlling chalkiness under high temperature stress were identified (Kobayashi et al. 2007; Tabata et al. 2007; Kobayashi et al. 2013; Wada et al. 2015; Miyahara et al. 2017). However, the genetic basis of chalkiness heat stress is still unclear. In Guandong province of China, rice is planted in two cropping seasons per year, FCS from late February to middle July and SCS from late July to middle November. During the seed filling period, the air temperature of FCS is usually higher than that of SCS. In 2016–2019, the mean temperature of FCS was 3.9 °C higher than that of SCS during the seed filling period (Additional file 1: Table S5). The PGC of HJX74, 11–09 and HP67–11 was 25.3%, 18.7% and 17.5% in FCS, and 16.7%, 6.4% and 5.2% in SCS, respectively. The PGC of all lines in FCS was higher than that in SCS at 0.001 level of significance (Fig. 5). Obviously, the grain chalkiness of HJX74 and SSSLs was greatly affected by higher temperature during the seed filling period. It is noted that the effect of high temperature on SSSLs, 11–09 and HP67–11, was greater than that on HJX74 (Fig. 5). We found that the additive effects of *qPGC9* and *qPGC11* on chalkiness in SCS were almost twice of those in FCS (Fig. 6). It indicates that the additive effects of *qPGC9* and *qPGC11* on chalkiness were decreased by the high temperature of FCS. The donor alleles of *qPGC9* and *qPGC11* were sensitive to high temperature. The results reveal the genetic basis of chalkiness heat stress. It laid a foundation for revealing the mechanism of chalkiness heat stress.

### Substitution mapping of QTLs for complex traits

Grain chalkiness is a complex polygenic quantitative trait and easily affected by environment (Sreenivasulu et al. 2015). Therefore, it is not easy to fine map QTLs for grain chalkiness. Chromosome segment substitution lines (CSSLs) carrying a small number of chromosome segments from one donor substituted into the genetic background of recipient are widely used in QTL analysis of complex traits (Howell et al. 1996; Doi et al. 1997; Kubo et al. 2002; Ebitani et al. 2005; Ebitani et al. 2008). When the CSSLs carry a single substitution segment from a donor, they are called SSSLs (Zhang et al. 2004; Xi et al. 2006), or NILs (Monforte and Tanksley 2000; Keurentjes et al. 2007). Through the development of SSSLs or NILs, the multiple QTLs of complex traits can be separated into a single QTL to accomplish the mendelization of QTL (Alonso-Blanco and Koornneef 2000). In the present study, the SSSLs with low grain chalkiness were selected to map the QTLs on the substitution segments. Through the development of secondary SSSLs, QTLs for grain chalkiness were limited in narrow intervals (Figs. 2 and 3). The SSSLs were tested for grain chalkiness in 8 cropping seasons of 4 years. The interaction of QTL by environment was detected in different environments of FCS and SCS (Figs. 5 and 6). The results indicate that substitution mapping is a powerful tool for the QTL analysis of complex traits.

## Conclusion

Two QTLs for grain chalkiness were located on two chromosomes. *qPGC9* was fine mapped on chromosome 9. *qPGC11* was fine mapped on chromosome 11. *qPGC11* is a new QTL for grain chalkiness from *O. glaberrima*. The effect of *qPGC9* and *qPGC11* was incomplete dominance. The additive effects of two QTLs on chalkiness in SCS were significantly greater than those in FCS. The donor alleles of *qPGC9* and *qPGC11* were sensitive to the high temperature of FCS. The results reveal the genetic basis of chalkiness heat stress in rice.

## Methods

### Plant materials and field experiments

Two SSSLs 11–09 and HP67–11 with lower grain chalkiness were selected from the SSSL library, in which HJX74, an *indica* variety of *O. sativa* in China was used as recipient. The substitution segment of 11–09 was derived from the donor Basmati 370, a Basmati variety of *O. sativa*. The substitution segment of HP67–11 was derived from the donor HP67, an accession of *O. glaberrima*. All plant materials were planted at the farm of South China Agricultural University, Guangzhou, China (23°07′N, 113°15′E) from 2016 to 2019. The materials were planted in two cropping seasons per year, FCS from late February to middle July and SCS from late July to middle November. Rice cultivation and controlling of diseases and insect pests were common practices in southern China.

### Measurement of grain chalkiness

The seeds of each line were harvested after full maturity. The dried seeds of 10 plants of each line were processed into milled rice and then 200 head rice of each plant were randomly selected for measurement of chalkiness. Images of the head rice were captured and the chalkiness parameters were measured by Microtek ScanWizard EZ scanner and rice quality analyzer SC-E software (Hangzhou Wanshen Detection Technology Co., Ltd., Hangzhou, China, www.wseen.com). PCG refers to the percentage of chalky grains in total grains. PCA refers to the percentage of chalk area per chalky grain. PGC is the product of percentage of chalky grains multiplied by percentage of chalk area.

### Genotyping of markers

Markers labeled “RM” were selected from online resources (https://archive.gramene.org/markers/). Markers of “PSM” and “InDel” were designed using the Primer Premier 5.0 software (Lalitha 2000) and the primer sequences of developed markers are listed in Additional file 1: Table S2. DNA samples were extracted from the fresh leaves of each plant by the method of Murray and Thompson (1980). DNA samples were amplified by PCR method. PCR products were separated by gel electrophoresis on 6% denatured PAGE and detected by the silver staining method (Tan et al. 2020).

### Phenotyping and statistical analysis

Heading date, plant height and panicle number per plant were investigated in the field. Grain traits were measured by the yield traits scorer (YTS), a rice phenotypic facility (Yang et al. 2014). The square root of the arcsine of the percentage was used for statistical analysis. The student’s *t*-test was used for comparison between two groups. Dunnett *t*-test was used to compare multiple groups with control group. Least significance range (LSR) was used for multiple range test among multiple groups (Duncan 1955). SPSS statistics 23.0 and Origin Pro 9.0 were used for data analysis and figure making (https://www.originlab.com).

### Substitution mapping of QTLs for PGC

To develop secondary SSSLs or NILs, SSSLs 11–09 and HP67–11 were crossed with the recipient HJX74. The NILs were developed from F_2:3_ populations derived from the crosses. The minimum, maximum and estimated lengths of a substitution segment were estimated by the positions of markers (Tan et al. 2020). The QTLs were located by substitution mapping (Eshed and Zamir 1995; Tan et al. 2020). When PGC showed significant difference between SSSL genotype and HJX74 genotype, a QTL for PGC was detected on the substitution segment of SSSL. When multiple substitution segments in NILs with target trait overlapped, the QTL was located in the overlapping region (Tan et al. 2020). Additive effect of a QTL was defined as the phenotypic difference between SSSL and HJX74 (Zhou et al. 2020). QTLs were named followed the method of McCouch et al. (1997). MapChart2.3 (https://www.wur.nl/en/show/Mapchart.htm) was used to draw the linkage maps of markers.

## Supplementary Information

**Additional file 1: Table S1.** Phenotypes of grain chalkiness in HJX74 and SSSLs. **Table S2**. Markers developed to detect the substitution segments of SSSLs. **Table S3.** Substitution segments of SSSLs. **Table S4.** Phenotypes of agronomic traits in SSSLs. **Table S5.** Average temperatures for 30 days after flowering of rice in two cropping seasons. **Table S6.** The grain shape of 11–09 and its NILs in the SCS of 2018. **Table S7.** The grain shape of HP67–11 and its NILs in the SCS of 2019.

**Additional file 2: Figure S1**. Frequency distribution of PGC in the F_2_ population derived from the cross of HJX74/11–09. *PGC* percentage of grain chalkiness. **Figure S2**. Frequency distribution of PGC in the F_2_ population derived from the cross of HJX74/HP67–11. *PGC* percentage of grain chalkiness.

## Data Availability

All data generated or analyzed in this study are included in this published article and its additional information files.

## Abbreviations

PGC: Percentage of grain chalkiness
PCG: Percentage of chalky grain
PCA: Percentage of chalky area
QTL: Quantitative trait locus
CSSL: Chromosome segment substitution line
SSSL: Single-segment substitution line
NIL: Near-isogenic line
FSC: First cropping season
SCS: Second cropping season
HJX74: Huajingxian 74
