# Supplementary material for: Fine mapping of two grain chalkiness QTLs sensitive to high temperature in rice
Source: Rice (N Y). 2021 Apr 1;14:33. doi: 10.1186/s12284-021-00476-x (PMC8017073; doi:10.1186/s12284-021-00476-x)
Supplement: Supplementary file 2 — Additional file 2: Figure S1. Frequency distribution of PGC in the F2 population derived from the cross of HJX74/11–09. PGC percentage of grain chalkiness. Figure S2. Frequency distribution of PGC in the F2 population derived from the cross of HJX74/HP67–11. PGC percentage of grain chalkiness. [file 12284_2021_476_MOESM2_ESM.docx]

**Additional file 2**





Figure S1. Frequency distribution of PGC in the F_2_ population derived from the cross of HJX74/11-09. *PGC* percentage of grain chalkiness.





Figure S2. Frequency distribution of PGC in the F_2_ population derived from the cross of HJX74/HP67-11. *PGC* percentage of grain chalkiness.
